# Controlling and modelling the wetting properties of III-V semiconductor surfaces using re-entrant nanostructures

**DOI:** 10.1038/s41598-018-21864-2

**Published:** 2018-02-23

**Authors:** Wing H. Ng, Yao Lu, Huiyun Liu, Claire J. Carmalt, Ivan P. Parkin, Anthony J. Kenyon

**Affiliations:** 1 Department of Electronic and Electrical Engineering, University College London, Torrington Place, London, WC1E 7JE United Kingdom; 2 Department of Chemistry, University College London, 20 Gordon Street, London, WC1H 0AJ United Kingdom

## Abstract

Inorganic semiconductors such as III-V materials are very important in our everyday life as they are used for manufacturing optoelectronic and microelectronic components with important applications span from energy harvesting to telecommunications. In some applications, these components are required to operate in harsh environments. In these cases, having waterproofing capability is essential. Here we demonstrate design and control of the wettability of indium phosphide based multilayer material (InP/InGaAs/InP) using re-entrant structures fabricated by a fast electron beam lithography technique. This patterning technique enabled us to fabricate highly uniform nanostructure arrays with at least one order of magnitude shorter patterning times compared to conventional electron beam lithography methods. We reduced the surface contact fraction significantly such that the water droplets may be completely removed from our nanostructured surface. We predicted the wettability of our patterned surface by modelling the adhesion energies between the water droplet and both the patterned surface and the dispensing needle. This is very useful for the development of coating-free waterproof optoelectronic and microelectronic components where the coating may hinder the performance of such devices and cause problems with semiconductor fabrication compatibility.

## Introduction

III-V semiconductors such as indium phosphide (InP) and gallium arsenide (GaAs) are very important class of materials for optoelectronics and high frequency microelectronics. Their use spans from lasers for telecommunications^1,2^ and imaging^3,4^, to photodetectors^5,6^, sensors^7^ and novel solar cells based on low dimensional structures^8,9^. Waterproofing is very important to protect such optoelectronics and electronics components embedded in consumer electronics such as mobile phone and laptops. Hydrophobic polymers such as polydimethylsiloxane (PDMS) are usually used to protect microchips from water, however, these polymers have poor thermal conductivity^10,11^, hindering heat dissipation^12^.

Waterproofing of III-V semiconductors has been reported previously, but previous studies involved transferring epitaxially grown III-V structures to flexible and waterproofing substrates such as PDMS^13^. This requires very complex fabrication procedures. As an alternative approach, surface nanostructuring has been demonstrated as an effective way to control hydrophobicity^14^ and suppression of ice formation^15^.

For fabrication of nanostructures, direct-write methods such as electron beam lithography (EBL)^16,17^ are some of the most popular techniques for patterning at the nanoscale. Although EBL can pattern features with resolutions of a few nanometres, and even sub-nanometre in some cases, conventional EBL takes a prohibitively long time to pattern large areas. While single-shot EBL strategies can improve the speed greatly^18–21^, most of the reported literature emphasizes the speed of the patterning itself rather than focusing on the quality of the resultant structures or their functionalities. In this paper, we demonstrate that by optimising the single shot beam dose with beam currents in the pico-Ampere (pA) range, we can achieve very highly uniform and high density multifunctional nanopillar arrays in a variety of materials, and on a variety of substrates over areas of square millimetres while maintaining a throughput of 10^6^ µm^2^ hr^−1^.

We also demonstrate that we are able to design surface morphologies to control wettability and make waterproof InP surfaces using the single shot EBL approach. We show that our highly uniform re-entrant (T-shaped) structures enable a highly water-repellent surface even without hydrophobic polymer treatment^22^, removing the need to work with organic materials that may not be compatible with semiconductor device processing. We support our experimental results with modelling of the surface adhesion energies of water droplets on water-needle and water-surface interfaces of the nanostructured surface; these models are in excellent agreement with our experimental results.

## Results and Discussion

### Fabrication of nanopillar arrays using FEBL method

**Figure 1:**
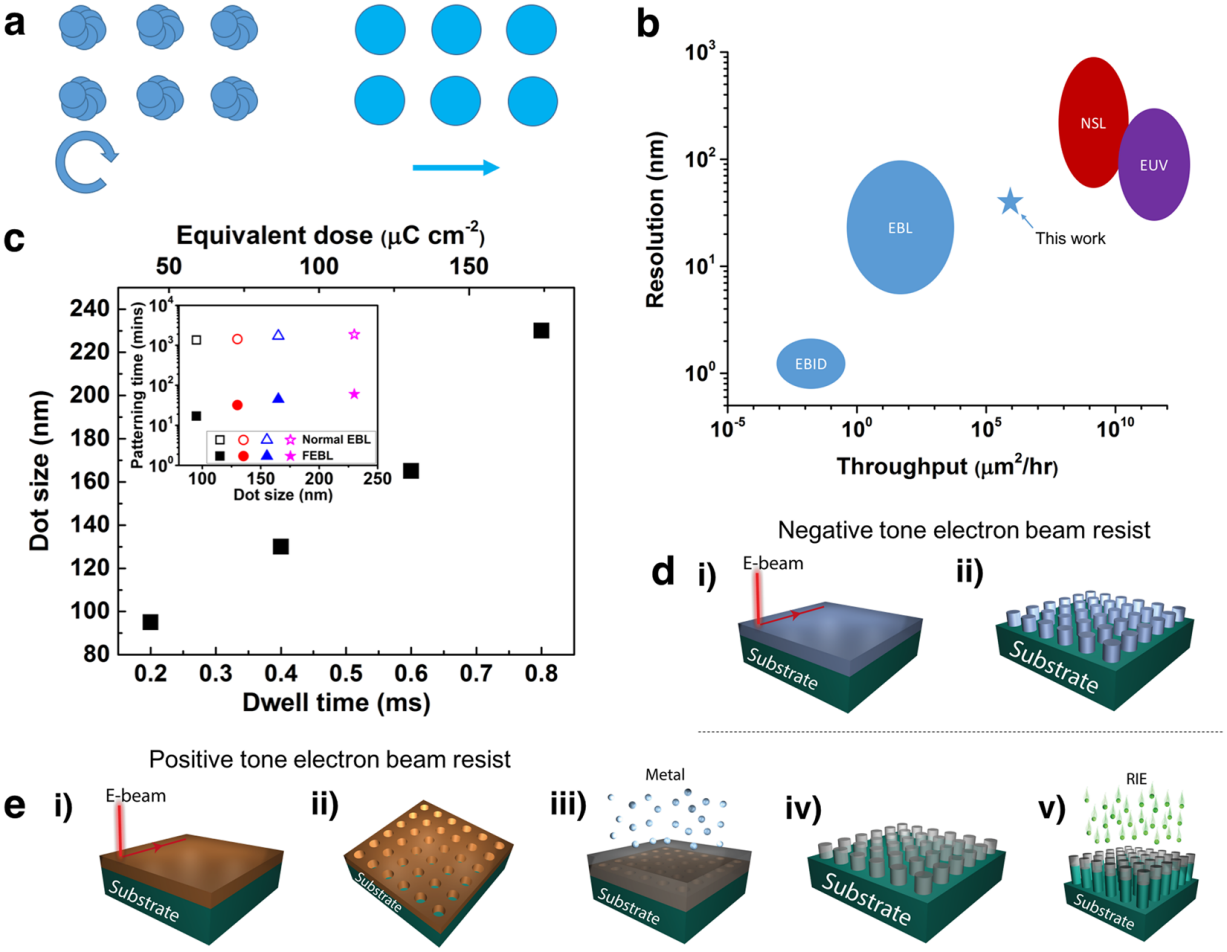
Fast Electron Beam Lithography (FEBL) patterning technique. (**a**) Patterning method for conventional e-beam lithography (left) vs. fast electron beam patterning technique (right). The former breaks down circular elements into multiple polygons; this pattern fracturing time contributes significantly to the total patterning time. In the FEBL patterning technique, the beam rasters continuously across the sample and dwells at specified points to define the spacing between adjacent elements. The diameter of the individual circular element is defined by the dwell time. (**b**) Resolution vs. throughput for selected nanolithography techniques. High throughput techniques such as nanosphere lithography (NSL) and current state-of-the-art photolithography (extreme ultraviolet EUV lithography) are wafer level techniques with very high throughput (over 10^9^ µm^2^/hr) with resolutions down to tens of nm. However, the pattern is pre-determined by the size of the nanosphere used in NSL and the optical mask for optical lithography. In contrast, direct write techniques such as EBL and electron beam induced deposition (EBID) offer much more flexible nano-patterning solution with sub-nm resolution. However, their throughput is limited to typically below 10^4^ µm^2^/hr (single beam system). The blue star indicates the throughput achieved by the FEBL technique for patterning nanodot arrays over millimetre-sized areas. (**c**) Dot (hole) size vs dwell time for the FEBL patterning technique applied to create PMMA holes on an InGaAs substrate. The array period was set to 500 nm. Over this interval the dot size increases roughly linearly with the electron beam spot dwell time. The equivalent beam dose scale is also provided. Inset, comparison of the patterning time for 1 mm × 1 mm array of nanoholes on PMMA with various hole diameters. The array period was 500 nm. The FEBL patterning technique is fully compatible with typical process flows for fabrication of nanodots and nanopillar arrays. (**d**) (i) Beam exposure on negative tone resist and (ii) Nanodot arrays formed after resist development. (**e**) (i) Beam exposure on positive tone resist. (ii) Nanoholes are left behind after resist development. (iii) and (iv) Metallic nanodots are fabricated after metal evaporation or sputtering followed by lift off. (v) We can also use the metal as etch mask for reactive ion etching (RIE) to fabricate high aspect ratio nanopillar arrays.

In contrast to conventional EBL, the “fast” (FEBL) method does not define individually the shape of the dots in our pattern design file. Instead, we specify the coverage area of the nanodot array and allow the electron beam to raster continuously across the sample surface, dwelling at specified intervals. These intervals define the centre-to-centre spacing of adjacent dots, and can be set independently in the x and y directions. By controlling dwell time, we vary the resist exposure, and consequently control the lateral diameter of the dot (Fig. 1a shows a schematic of the beam rastering). As a result, highly uniform nanodot arrays with specified spacing and dot size can be obtained. Significantly, the patterning time for this method is much shorter than conventional EBL since the beam continuously rasters and dwells rather than drawing individual circles as multiple polygons (Illustrated in Fig. 1a). Using conventional EBL resists we can obtain a patterning throughput greater than 10^6^ µm^2^ hr^−1^ with this method.

Figure 1b shows how the FEBL technique compares with other standard micro- and nanolithography techniques, Mask-based techniques are dominated by photolithography; although this has extremely high throughputs with resolutions of tens of nanometres achievable using EUV sources, the pattern is predetermined by the mask, hence is not flexible. Colloidal or nanosphere lithography is capable of large area (>cm^2^) array patterns using self-assembled nanosphere masks, and provides some degree of patterning flexibility. However, while the most flexible techniques are direct-write methods such as electron beam lithography and electron beam induced deposition (EBID)^23^ provide complete control of pattern size and shape, they suffer from low throughput and are hence not ideal for large area (mm^2^) patterning. While conventional EBL would take more than 24 hours to generate a 1 mm × 1 mm array of 300 nm diameter dots in a 2D square lattice with a dot-to-dot spacing of 500 nm, the FEBL method greatly reduces the required time to 1 hour. We thus combine the flexibility of direct-write methods with speed comparable with optical lithography.

**Figure 2:**
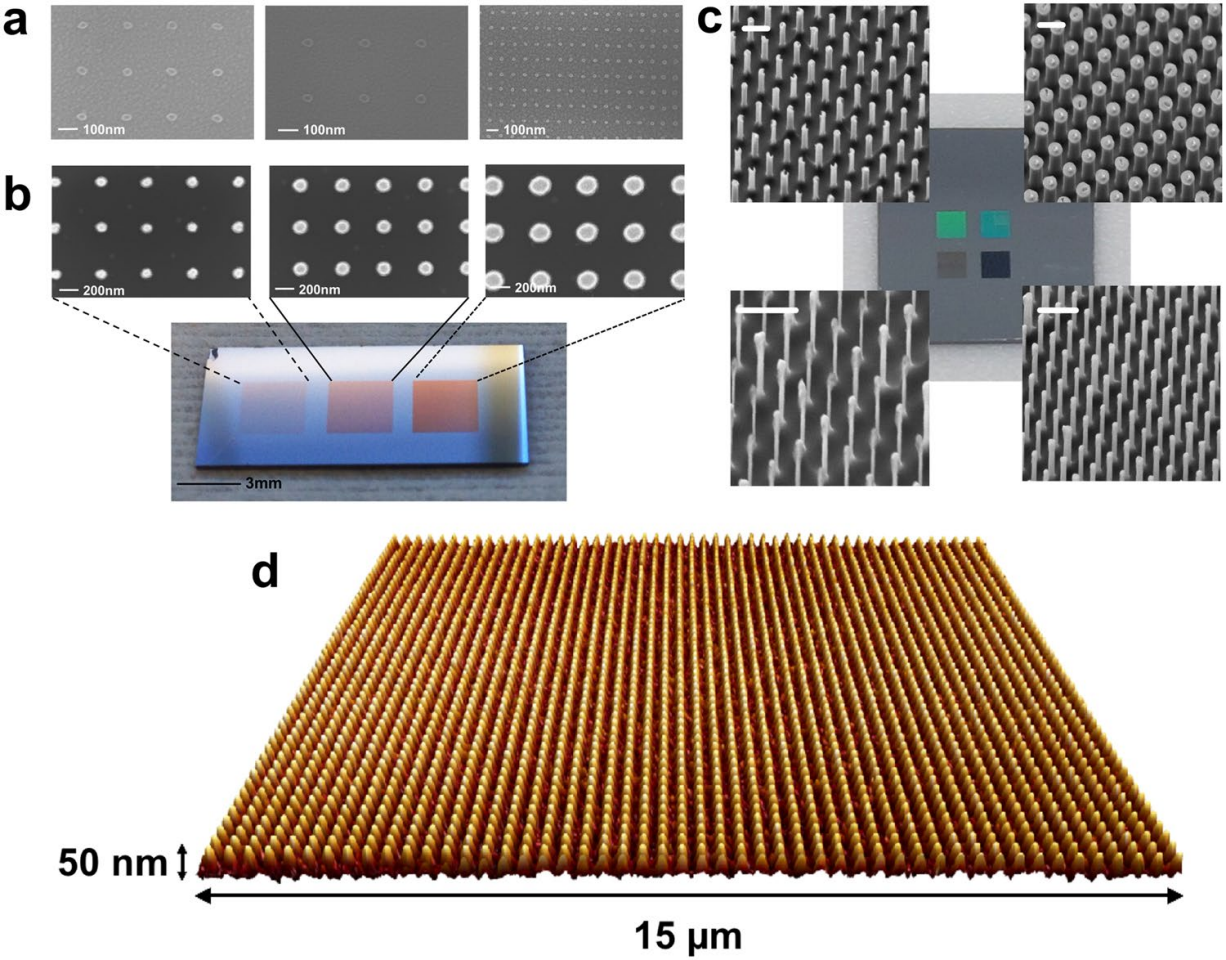
SEM, AFM and optical images of nanoarrays. (**a**) Scanning electron microscope (SEM) images of the HSQ nanodot arrays (height and diameter = 30 nm) fabricated on an ITO coated SiO_2_/Si substrate. The array period varies from 200 nm (left image), 300 nm (middle image) to 90 nm in the x-direction and 120 nm in the y-direction (right image). (**b**) Optical image of three sets of 3 mm × 3 mm gold nanodot arrays fabricated on a Si substrate. The array period is fixed at 500 nm. The SEM images show the corresponding diameters of the gold nanodots: 110 nm (left image), 170 nm (middle image) and 210 nm (right image). The optical image shows a difference in reflectivity intensities due to the difference of the fill factor of the gold nanodot array. (**c**) Optical image of four sets of 1.5 mm × 1.5 mm Si nanopillar arrays fabricated on a Si chip. The height (H) of the nanopillars is 950 nm. The optical reflectivity of the Si surface can be adjusted by the pillar diameter and array period. The SEM images show the corresponding array dimensions – bottom left image, array period (L) = 500 nm, diameter (D) = 110 nm; bottom right image L = 500 nm, D = 160 nm; top left image L = 1 µm, D = 280 nm, top right image L = 1 µm, D = 570 nm (scale bar = 1 µm). (**d**) Atomic force microscopy (AFM) image of a 15 µm × 15 µm HSQ nanopillar array, illustrating the uniformity of the arrays fabricated by the FEBL technique.

**Figure 3:**
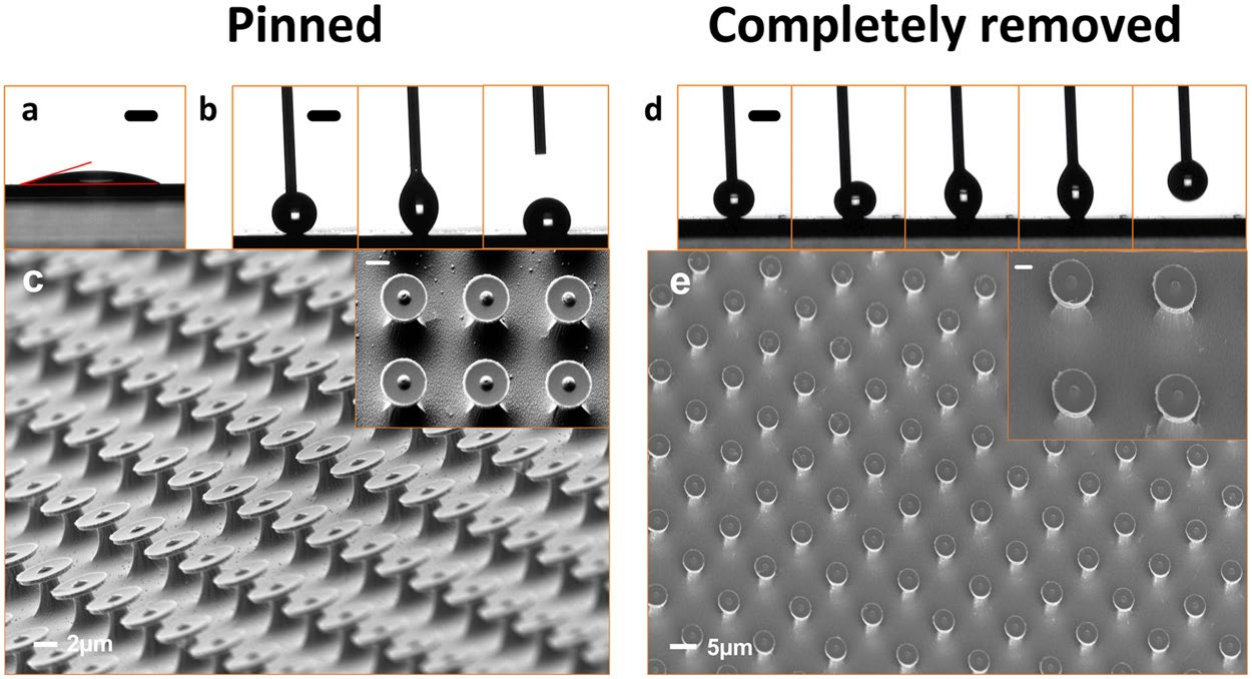
Designing surface morphologies to make water repellent surfaces. (**a**) Water droplet on a flat InP wafer, which is readily wetted (contact angle = 22.4°), indicating its hydrophilicity (scale bar = 1 mm). (**b**) Sample A1: Water droplet contacts a textured InP surface with re-entrant structures (D = 4.5 μm, L = 8 μm, H = 2.5 μm, λ_1_ = 0.249, where λ is the fraction of the solid in contact with the liquid); the water droplet was eventually pinned on the surface (contact angle = 124.5°). (**c**) SEM images of the designed surface used in b; insert shows a top view of the pillars (scale bar = 2 µm). (**d**) Sample A2: Water droplet contacts a designed InP surface with re-entrant structures (D = 4 μm, L = 10 μm, H = 2 μm, λ_2_ = 0.126). A smaller contact fraction enables the water droplet to be removed completely from the surface (scale bar = 1 mm). (**e**) SEM images of the designed surface used in d. Insert shows a top view of the pillars (scale bar = 2 µm). (Water droplet size in all cases: ~2 μL).

Figure 1c illustrates how the exposed dot (hole) size on a poly-methyl-methacrylate (PMMA) layer on InGaAs varies with the dwell time at each spot. The EBL used was a Raith-150 TWO system. The beam energy used was 30 kV with beam current set to 540 pA. The spacing between dwell spots was set to 500 nm. The graph shows that by varying the dwell time from 0.2 to 0.8 ms, which is equivalent to varying the beam dose from 40 to 175 µC cm^−2^, the dot size varies from 95 nm to 230 nm. As seen below, we can decrease the dose in order to create smaller diameter dots (Fig. 2b). On the other hand, we can increase the dwell time to hundreds of milliseconds in order to write larger structures, as shown in Fig. 3. The inset in Fig. 1c shows the patterning time for a 1 mm × 1 mm array of nanodots with various dot diameters using the same substrate (PMMA on InGaAs). The array period (centre to centre spacing between adjacent dots) was set to 500 nm. For an array of 95 nm dots, the patterning time under normal EBL mode is over 1300 mins. By contrast, in the FEBL mode, the patterning time is less than 20 mins, equivalent to a throughput >10^6^ µm^2^ hr^−1^. This reduction in patterning time of nanodots by around two orders of magnitude comes from the fact that the EBL system usually needs time to fracture the circle that defines the dot area into polygons before exposure. In the FEBL mode, this fracturing time is reduced to close to zero since the circle is defined by a single dot, and the main contribution to the patterning time is the beam dwell time during writing. Although there are literature reports of greater throughput using higher beam dose^18,19^, the uniformity of the arrays is compromised as a result. While there is an inevitable trade-off between uniformity and speed, we demonstrate here that we can optimize the quality of the structures while maintaining high throughput.

This technique is fully compatible with existing top-down fabrication schemes using both positive and negative tone photoresists (Fig. 1d,e). To demonstrate the flexibility and controllability of our FEBL technique, we fabricated a series of hydrogen silsesquioxane (HSQ, a negative tone electron beam resist) nanodot arrays on an indium tin oxide (ITO)-coated silicon oxide/silicon substrate (Fig. 1d). The SEM images in Fig. 2a show an example sample in which dot diameter is fixed at 30 nm, with the pitch varying from 200 nm (left image) to 300 nm (middle image). The right image of Fig. 2a demonstrates that the x and y pitches can be controlled independently. These examples demonstrate the versatility of the FEBL method in controlling the size and spacing of the nanoarray.

Figure 2b shows gold nanodots fabricated on a silicon substrate by exposing the nanoarray pattern onto poly-methyl methacrylate (PMMA), a positive tone resist, followed by gold thin film deposition and lift-off in acetone (Fig. 1e, i to iv). Figure 2b shows the corresponding optical image of the gold nanodot arrays deposited on a Si substrate. Each array is 3 mm × 3 mm and the pitch between dots is 500 nm. By varying the beam dwell time we can fabricate arrays with dot sizes of 110 nm, 170 nm and 210 nm.

We can also go one step further and use the patterned metallic dots as etch masks for the fabrication of nanopillar arrays (Fig. 1e, iv to v). The centre optical image in Fig. 2c shows a white light optical reflection image of four such 1.5 mm × 1.5 mm sets of 950 nm tall Si nanopillar arrays with different diameters and array pitches. The reflected colours vary with pillar dimensions – light brown, green, blue and black (details in the figure caption). The corresponding SEM images of the pillars are next to the optical images. All four squares were patterned in the same run, demonstrating that nanodot and nanopillar arrays with mixed dimensions can be made simultaneously in the same sample.

The uniformity of our arrays over large areas is excellent. The atomic force microscope (AFM) scan in Fig. 2d shows a representative 15 µm × 15 µm area of a 3 mm × 3 mm array of HSQ nanopillars in which the variation of the feature height and pitch is less than 2%. A large write field was used (500 µm × 500 µm) to minimize the effect of stitching errors between write-fields. AFM scans of a number of regions within the whole sample area demonstrated similar uniformity.

Once we had established the practicality of the FEBL method to pattern large areas, in order to demonstrate how the control of surface morphology at the nanoscale can control hydrophobicity, we fabricated arrays of re-entrant structures tailored to maximise the hydrophobicity of surfaces. Our samples were fabricated from indium phosphide/indium gallium arsenide (InP/InGaAs) multilayer thin films grown by molecular beam epitaxy on InP substrates. We used the FEBL technique to pattern the surface, and subsequent reactive ion etching and wet etching form the re-entrant structures on the top InP surface (See Methods).

### Using the single shot EBL technique to fabricate superhydrophobic surfaces

Before we fabricated the re-entrant structures, we tested the wettability of the InP substrate using contact angle measurement. Figure 3a shows a water contact angle of ~22.4° on a plain InP layer surface, indicating that without surface treatment, it is hydrophilic.

We fabricated two sets of re-entrant structures for this study, Fig. 3b shows a water droplet contacting Sample A1, with has arrays of mushroom-shaped pillars of diameter (D) = 4.5 µm, array period (L) = 8 µm, and height (H) = 2.5 µm; the water droplet was finally pinned onto the surface with a contact angle of 124.5°, indicating its hydrophobicity (supplementary movie S1). We modelled the contact angle of the designed surface (see the analysis section), obtaining a value of 121.4°, in excellent agreement with the measured value. Figure 3c shows a scanning electron microscope (SEM) image of the resultant structures, which were fabricated over an area of 3 mm × 3 mm.

We next designed a surface that would allow a water droplet to be completely removed from the surface (Sample A2), and again fabricated this by the FEBL technique. This structure has a dimensions D = 4 μm, L = 10 μm, H = 2 μm, and using this design we are able to completely remove the water droplet from the surface using the capillary needle, as shown in Fig. 3d and supplementary movie S2. Figure 3e shows SEM images of the surface morphology of the sample. Such superhydrophonic surfaces were inspired by the ‘rose petal effect’^24–28^, or gecko hand^29–32^, in which the surfaces have water contact angles greater than 150 degrees with a high hysteresis. These results show that the single shot EBL surface patterning technique allow us to design the surface contact fraction in order to remove or pin the water droplet on the substrate surface, without requiring the addition of hydrophobic polymer coatings.

### Modelling the adhesion energies of the water droplet on water-surface interfaces

We created the mushroom-like features on hydrophilic surfaces so they would in part protect the surface from wetting in a Cassie-Baxter state^33^, in which case the contact angle of the water droplet with the structured surface can be written as:

1 $$\cos \,\theta * =-1+\lambda (1+\,\cos \,\theta )$$

where θ* is the apparent contact angle, *θ* is the inherent contact angle (*θ* = 22.4° in the case of a flat InP substrate), and *λ* is the fraction of the solid in contact with the liquid. Take Sample A1 as an example, *λ*_1_ can be calculated from the designed pillar dimensions of D and L (D = 4.5 µm and L = 8 µm), assuming the whole water droplet was suspended by the re-entrant structures without touching the bottom of the substrate. In this case, as shown in Fig. 3b and c, *λ*_1_ = 0.249. Using equation (1), θ* was calculated to be 121.4°, which is in good agreement with the measured value (124.5°).

In order to design a surface that allows a water droplet to be completely removed from the surface, we calculated the adhesion energy W at the water-needle (*W*_*n*_), and the water-substrate interfaces (*W*_*s*_) (Fig. 3d), given by the Young-Dupre equation^34,35^:

2 $$W=\gamma (1+\,\cos \,\theta * )A$$

*γ* is the surface tension of water (71.97 mJ/m^2^), *A* is the apparent interface area between the water droplet and the contact surface.

In order to completely remove the water droplet from the surface, the adhesion energy between the dispensing needle and water (*W*_*n*_) must be greater than sum of the adhesion energy between the water and substrate (*W*_*s*_) and the energy required to lift the droplet against its own weight (*E*_*L*_) i.e. *W*_*n*_ > *W*_*s*_ + *E*_*L*_, Fig. 3d.

### Calculation of adhesion energy between the dispensing needle and water (*W*_*n*_)

**Figure 4:**
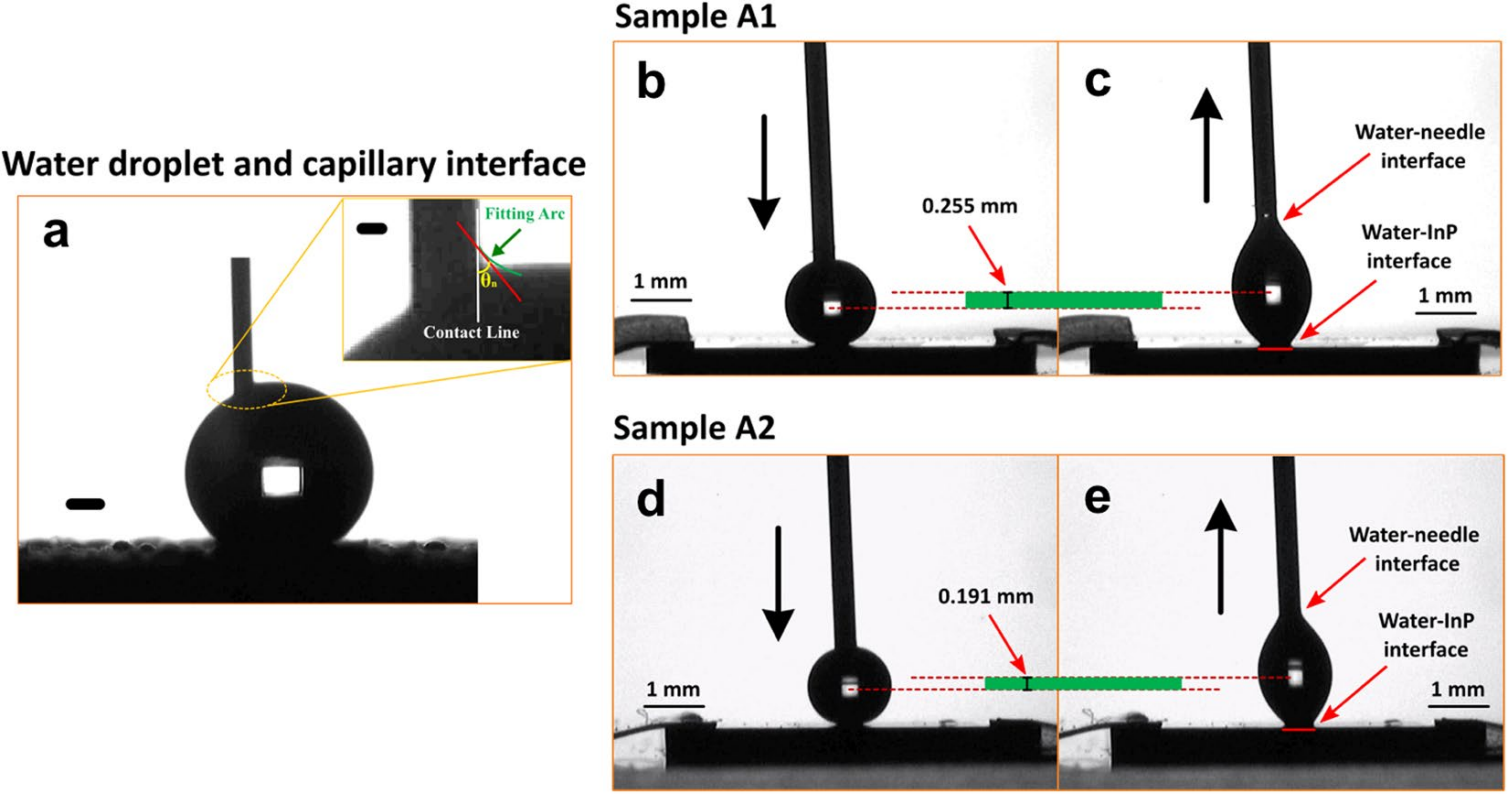
Estimation of adhesion energies among the interfaces of water droplet, dispensing needle and re-entrant structure surfaces. Interface between the water droplet and the capillary needle. (**a**) The syringe needle was inserted into a water droplet that was supported by a hydrophobic surface (Bars: 1 mm; insert, 100 μm). The contact angle of the needle surface was measured through fitting an arc of the meniscus of water and then fitting a tangent line at the intersection of the water-needle contact line and the fitted arc, the angle *θ*_*n*_ between the contact line and the tangent line is the contact angle of the needle surface. Water droplet contacting and leaving the surface of the Sample A1. (**b**) The needle was lowered to allow the water droplet contact the substrate. (**c**) The needle was raised and tended to remove the water droplet from the substrate. It showed a hysteresis due to the hydrophilicity of the InP surface. The red line at the water-InP interface shows the diameter of the contact area when the droplet was about to leave (*D*_s1_ = 0.486 mm). Comparing b and c, the barycentre of the water droplet rose by 0.255 mm. Water droplet contacting and leaving the surface of the Sample A2. (**d**) The needle was lowered to allow the water droplet contact the substrate. (**e**) The needle was raised and tended to remove the water droplet from the substrate. It showed a hysteresis due to the hydrophilicity of the InP surface. The red line at the water-InP interface shows the diameter of the contact area when the droplet was about to leave (*D*_s2_ = 0.467 mm). Comparing d and e, the barycentre of the water droplet rose by 0.191 mm.

The adhesion energy at the water-needle interface (*W*_*n*_) consists of two components: the energy between the water-water contact at the centre of the needle tip (*W*_*w*_), and the energy between the water-needle contact at the rim of the needle (*W*_*r*_):

3 $${W}_{n}={W}_{w}+{W}_{r}=2\gamma {A}_{w}+\gamma (1+\,\cos \,{\theta }_{n}){A}_{r}$$

where *A*_*w*_ is the area of water-water contact at the tip of the needle, and *A*_*r*_ is the area where the water contact at the rim of the needle. *θ*_*n*_ is the water contact angle of the needle surface, measured as shown in Fig. 4a.

According to the Young-Dupre equation (equation 2), *W*_w_ = 2*γA*_w_, and *W*_r_ = *γ*(1 + cos *θ*_*n*_) *A*_r_. Here, *A*_*w*_ = π(d_i_/2)^2^, d_i_ is the inner diameter of the needle; *A*_r_ is the area where the water contacts the surface of the needle, in the extreme case of water released from the needle, this area is the bottom area of the needle and can be calculated as *A*_r_ = π(D_o_/2)^2^ − π(d_i_/2)^2^, where D_o_ is the outer diameter of the needle. The needle is of 30 gauge with outer and inner diameters of D_o_ = 0.3112 mm and d_i_ = 0.159 mm, respectively, then *A*_w_ = 0.0199 mm^2^ and *A*_r_ = 0.0562 mm^2^. We measured this *θ*_*n*_ using a hydrophobic surface as shown in Fig. 4a. The needle was inserted into the water droplet, the meniscus between the needle and water interface demonstrated the contact angle of the needle (39.6°)^36^. It then can be obtained that *W*_w_ = 2.864 × 10^−6^ mJ and *W*_r_ = 7.163 × 10^−6^ mJ. Then *W*_n_ = *W*_w_ + *W*_r_ = 1.0027 × 10^−5^ mJ.

### Calculation of adhesion energy between water and Sample A1 substrate (*W*_s1_)

The adhesion energy between the water droplet and the substrate for our Sample A1 (*W*_s1_) can be calculated by combing equations (1) and (2),

4 $${W}_{s1}=\gamma {\lambda }_{1}(1+\,\cos \,\theta ){A}_{s1}$$

where *A*_s1_ is the area of the apparent interface between the water droplet and the InP substrate (the area is a circle with a diameter of *D*_s1_ = 0.486 mm as shown in Fig. 4c). Therefore, *A*_s1_ = π(*D*_s1_/2)^2^ = 0.1855 mm^2^. Given λ_1_ = 0.249 and θ = 22.4°, *W*_s1_ = 6.398 × 10^−6^ mJ.

### Energy required to lift the water droplet by the needle in Sample A1 (*E*_*L1*_)

The energy required to lift the droplet by the needle (*E*_*L*_) is equivalent to the work done on lifting the droplet vertically away from the surface. Work done can be expressed as:

5 $${E}_{L}=M\times G\times B$$

*M* = mass of water droplet, *G* = gravitational acceleration = 9.8 m/s^2^, *B* = maximum distance of the barycentre moved when lifting the droplet before it is removed from the sample surface or needle.

The volume of the droplet used was 2 µL, which has a mass of 2 × 10^–6^ kg. For sample A1, the distance of the barycentre moved was 0.255 mm (see Fig. 4b,c). From these numbers we estimated *E*_*L1*_ to be 4.998 × 10^−6^ mJ.

For sample A1, the water-needle adhesion energy (*W*_*n*_ = 1.0027 × 10^−5^ mJ) is less than the sum of the water-substrate adhesion energy and energy used to lift the droplet (*W*_*s1*_ + *E*_*L1*_ = 1.1396 × 10^−5^ mJ). As a result, the water droplet was pinned onto the substrate surface when attempting to lift it up by the needle.

### Calculation of adhesion energy between the water and Sample A2 substrate (*W*_s2_)

Sample A2 was fabricated using our FEBL technique with *λ*_2_ = 0.126 (D = 4 μm, L = 10 μm, H = 2 μm). Based on equation (4), *W*_s2_ = *γ λ*_2_ (1 + cos *θ*) *A*_s2_, where *A*_s2_ is the area of the apparent interface between the water droplet and the InP substrate (the area is a circle with a diameter of *D*_s2_ = 0.467 mm as shown in Fig. 4e). Therefore, *A*_s2_ = π(*D*_s2_/2)^2^ = 0.1713 mm^2^. Given λ_2_ = 0.126 and θ = 22.4°, *W*_s2_ = 2.990 × 10^–6^ mJ.

### Energy required to lift the water droplet by the needle in Sample A2 (*E*_*L2*_)

Using equation (5), with M = 2 × 10^–6^ kg and G = 9.8 m/s^2^, for sample A2, the distance the barycentre moved was 0.191 mm (see Fig. 4d,e). From these numbers we estimated *E*_*L2*_ = 3.744 × 10^−6^ mJ.

In this case the water-needle adhesion energy (*W*_*n*_ = 1.0027 × 10^−5^ mJ) is greater than the sum of the water-substrate adhesion energy and energy required to lift the droplet (*W*_*s2*_ + *E*_*L2*_ = 6.7329 × 10^−6^ mJ), therefore sample A2 satisfied the condition *W*_*n*_ > *W*_*s*_ + *E*_*L*_ and the water droplet could be removed completely.

## Conclusion

In summary, we demonstrated the design and fabrication of hydrophobic surfaces on intrinsically hydrophilic III-V materials using re-entrant structures. The FEBL method was able to produce highly uniform nanostructure arrays with much reduced patterning time, and the excellent uniformity of the fabricated array enables the demonstration of hydrophobicity in our III-V material. By changing the size and spacing of the nano- and micro-structures, we are able to control the wettability of the surfaces, and in optmised cases water can be completely removed from the surface. The wettability of our re-entrant surfaces can be predicted by modelling the adhesion energies between the water droplet and the patterned surface. This provides a simpler route for integrating waterproofing features into optoelectronic components compared to enclosing the component in polymer coatings, which may be incompatible with the harsh environmental conditions required for semiconductor device processing, such as high temperatures.

## Methods

### Fabrication methods for the InP/InGaAs re-entrant (T-shaped) structures

The re-entrant micro-structures were fabricated on a 1 cm × 1 cm InP/InGaAs multilayer thin film grown by molecular beam epitaxy and lattice matched to an InP substrate. The top InP layer was 300 nm thick and a 3 µm InGaAs layer was sandwiched between the top InP layer and bottom InP substrate. Such a structure is typical of many optoelectronic devices.

A 300 nm layer of PMMA was spin-coated on the top InP layer and the array pattern was written onto the PMMA layer by Fast Electron Beam Lithography (Raith-150 TWO 30 kV system). The beam was set to dwell every 8 µm for sample A1 and every 10 µm for sample A2. Dwell time and beam energy was adjusted to define the final size of exposed spot, which in turn defined the size of the top of the re-entrant structure. The patterned area was 3 mm × 3 mm. Because the FEBL technique does not require dithering to generate patterns – only step and dwell – we were able to pattern large areas in extremely short times. After resist development in MIBK:IPA solution, 100 nm of Cr was deposited on top of the sample by thermal evaporation (Edwards A500) followed by lift off in acetone. This formed an array of Cr microdisks on the InP layer which served as an etch mask. The sample was then etched through to the InGaAs layer by reactive ion etching (RIE) using a CH_4_ and H_2_ plasma. After RIE, hydrofluoric acid (HF) solution was used to selectively under-etch the InGaAs layer. As a result, a T-shaped microdisk array was formed for wettability studies (Fig. 3c,e).

### Sample characterisation

The SEM images were taken using Zeiss XB1540 CrossBeam focused ion beam system. The system has a built-in electron beam column, in which the images were taken with an accelerating voltage of 5 kV.

### Contact angle measurements

The water contact angles (including images and videos taking, see supplementary videos) were measured at ambient temperature via the sessile-drop method using an optical contact angle meter (FTA 1000, water droplet is ~2 μL).

### Data availability

All data generated or analysed during this study are included in this published article (and its Supplementary Information files).

## Electronic supplementary material

Supplementary Movie S1

Supplementary Movie S2
